# Dispersion curve engineering for automated topology design of a unit cell in spoof surface plasmon polaritons

**DOI:** 10.1038/s41598-024-52842-6

**Published:** 2024-02-18

**Authors:** Salma Mirhadi, Zahra Javidi, Nader Komjani

**Affiliations:** 1 Department of Electrical and Computer Engineering, Shariaty College, Technical and Vocational University, Tehran, Iran; 2 Department of Electrical Engineering, Iran University of Science and Technology, Tehran, Iran

**Keywords:** Engineering, Electrical and electronic engineering

## Abstract

In this paper, an automatic design method is proposed for unit cell in spoof surface plasmon polaritons (SSPP) with an almost arbitrary dispersion curve. In this method, the pixel configuration is considered for the unit cell and, by using the binary particle swarm optimization method, the proper topology of the unit cell is explored so as to reach the target dispersion curve. Unlike the traditional method of controlling the dispersion curve, which is performed based on changing the geometric parameters of the predetermined unit cell, in this method, there is no need to know the shape of the unit cell, and the dispersion curve of the modes of SSPP unit cell can be controlled independently with more freedom. Two unit cell samples are designed in order to show the efficiency of the procedure. In the first sample, the dispersion curve is designed to have the lowest asymptotic frequency; in the second sample, the dispersion curve of the second mode is controlled independently from the first mode and is changed arbitrarily. SSPP transmission lines which are related to the unit cells of the two samples are designed, and it is demonstrated that measurement and simulation results are greatly in line with each other.

## Introduction

Surface plasmon polaritons (SSPs), which arise from the coupling between electromagnetic waves and free electrons of metals at optical frequencies, propagate along the metal–dielectric interface and decay exponentially on both sides in the direction perpendicular to the boundary^1^. By overcoming the diffraction limit in the sub-wavelength scale, SPPs are concentrated at the interface between two materials providing the possibility of minifying the size of optical integrated circuits and devices^2–5^. The intrinsic oscillation of metal electrons at frequencies higher than infrared occurs from their plasma type behavior and the creation of a negative electrical conductivity coefficient. At lower frequencies, such as terahertz and microwaves, metals behave as perfect conductors and SPP is not realized. However, during the past decades, scientists have achieved SSP-like dispersion relations and high field confinement in the sub-wavelength scale at lower frequencies, by introducing spoof surface plasmon polaritons (SSPPs) consisting of periodic metal arrays with grooves, holes, or slits^6–11^. The dispersion relation of the TM-polarized SSPP waves propagated in a metallic groove array with infinite thickness is expressed by^7^:

1 $$ k = k_{0} \sqrt {1 + \frac{{a^{2} }}{{p^{2} }}{\text{tan}}^{2} \left( {k_{0} h} \right)} $$

where $p$, $a$, and $h$ are the period, width, and the depth of grooves, respectively. $k_{0}$ and $k$ is the wave number of EM waves in free space and along the propagation direction, respectively. Although the dispersion diagram of the unit cell of these periodic arrays can be controlled by changing its geometric parameters, the adjustability of the dispersion curve is done in a limited way by keeping the original shape of the unit cell and changing its dimensions; it is not necessarily possible to ensure that the response is optimized. On the other hand, although efforts have been made to derive the dispersion relation for more complicated grooves^12,13^, it is impossible to get an analytical expression for the SSPP waveguides with irregular-shaped grooves. The only thing that can be said with certainty is that the shape of the grooves affects the dispersion curve and the field confinement. For instance, in 2019, the unit cell with fence-shaped grooves was proposed as a unit cell that has higher field confinement than the previously presented unit cells^14^. But whether there is a unit cell with higher concentration is not known yet. Besides, sometimes by changing the geometrical parameters of the unit cell, it is not possible to obtain a specific dispersion curve or separate control of higher modes. For instance, in 2020, a waveguide of complementary spoof surface plasmon polaritons (CSSPPs) including groove slots loaded with interdigital structure (IS) slabs were proposed and, by increasing the number of IS slabs, cutoff frequency of the dispersion curve of the first mode decreased, but there is a question whether it is possible to change the dispersion curve of the second mode without changing the dispersion curve of the first mode, or not^15^. Consequently, choosing the predetermined unit cells in traditional designs and changing or even optimizing their geometric parameters has limitations in changing the dispersion curve and does not provide enough freedom to reach the desired dispersion curves. In contrast, by optimizing the shape of the unit cell and using topology engineering, the dispersion diagram can be changed more freely so as to design various SSPP TLs and realize different applications.

So far, such an approach has been used in designing the shape of various structures such as antennas^16–18^, metasurfaces^19–22^ and frequency selective surfaces^23^ using optimization and pixel shape extraction methods. In the present study, given the aforementioned aims, the pixel design strategy is used for the unit cells of SSPP structures for the first time.

## Designing method

**Figure 1:**
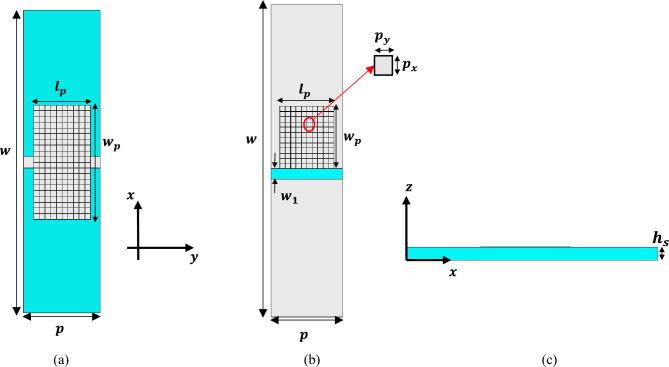
(**a**) SSPP and (**b**) CSSPP unit cell, with a part of its surface pixelated. The blue color represents the substrate and the gray color represents the metal. In the SSPP unit cell, the pixelation is done bilaterally, but in the CSSPP unit cell, the pixelation is done unilaterally. In order to reduce the dimensions of optimization, the unit cell can be considered to have two-sided or even four-sided symmetry. *p* is the periodicity of the cell, *p*_*x*_ and *p*_*y*_ are the pixel dimensions in the x and y directions, respectively, (**c**) side view of the structure.

The substrate used in the designs of this article is RO4003 with thickness of *h*_*s*_ = 0.8 mm and relative dielectric constant of 3.55. A copper layer with thickness of 35 μm is also used as a conductor. In order to design the topology of the unit cell, a part of its surface is pixelated, as shown in Fig. 1. In SSPP structures (Fig. 1a), if each pixel is 1, it shows the presence of metal; if it is 0, it shows the absence of metal; in CSSPP structures (Fig. 1b), if each pixel is 0, it means the metal is present; and 1 indicates the absence of metal. The general shape of the unit cell is obtained from juxtaposing pixels, which can be considered as a binary string. Every pixel plays a role in determining the dispersion curve. So as to obtain the intended dispersion curve, the proper binary string must be determined using one of the discrete optimization methods with the binary solution space.

One of the binary optimization methods which has been greatly noticed in recent years in order to design the shape of structures is the binary particle swarm optimization (BPSO) method. In this method, a group of particles move in the N-dimensional space of the problem and search for the best solution. In each iteration, particles adjust their location and velocity based on their previous best experience and the location of the group's previous best experience. In the discrete solution space, transfer functions such as the sigmoid function are used to link the continuous values of particle velocity to the discrete values of particle location^24^. Owing to the successful performance of this method in pixel designs, this method is also chosen in this article.

**Figure 2:**
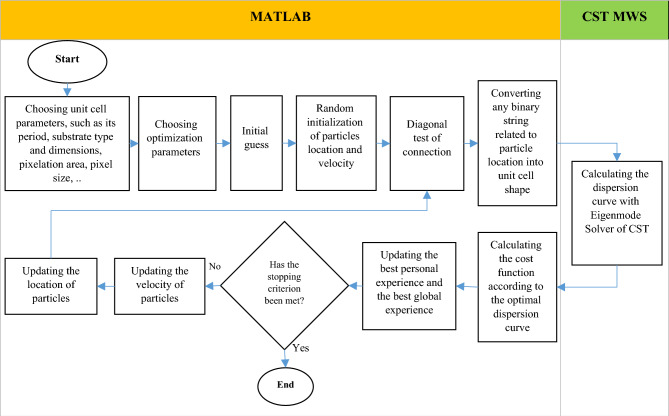
Flowchart of automatic design of the unit cell topology.

The flowchart of Fig. 2 is drawn so as to illustrate the main parts of automatic design stages of the unit cell shape to realize the intended dispersion curve. In this design, it is necessary to link a full electromagnetic wave simulator in order to calculate the dispersion curve for each binary string (each unit cell topology) and a programming environment to implement the optimization algorithm. Electromagnetic analyzer program, CST Microwave Studio (CST MWS) software and MATLAB programming environment are selected. For optimization, an initial guess is required for the answer. This initial guess can be obtained by using the articles presented so far. Besides, the optimization cost function, which is a criterion for stopping the optimization, is defined according to the intended dispersion curve.

**Figure 3:**
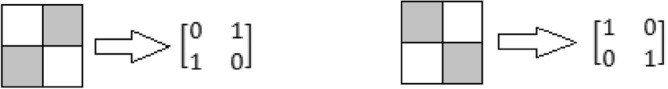
Two inappropriate point connections and its corresponding matrix.

One of the issues that should be taken into account in this type of design due to construction considerations is to prevent connection at one point. This type of point connection which is represented in Fig. 3 is created when the diagonal matrix of the connection is in the form $$\left[ {\begin{array}{*{20}c} 1 & 0 \\ 0 & 1 \\ \end{array} } \right]$$ or $$\left[ {\begin{array}{*{20}c} 0 & 1 \\ 1 & 0 \\ \end{array} } \right]$$. This type of connection is problematic in practice and in measurement results because theoretically there is no connection between two pixels, while physically there is. So as to solve this problem, first of all, this type of connection is identified in each binary string and, then, it is modified as follows: first, a random number, like r, is selected in the (0, 1) interval. If this number is in the (0, 0.5) interval, the matrixes are turned into $$\left[ {\begin{array}{*{20}c} 1 & 1 \\ 1 & 0 \\ \end{array} } \right]$$. However, if this number is in the [0.5, 1) interval, the matrixes are turned into $$\left[ {\begin{array}{*{20}c} 0 & 1 \\ 1 & 1 \\ \end{array} } \right]$$. Also, due to increasing the speed of the program and reducing the dimensions of the optimization problem, two- or four-sided symmetry can be used in the structure of the unit cell. After selecting the optimization and unit cell parameters, the program continues automatically to achieve the desired dispersion curve.

**Figure 4:**
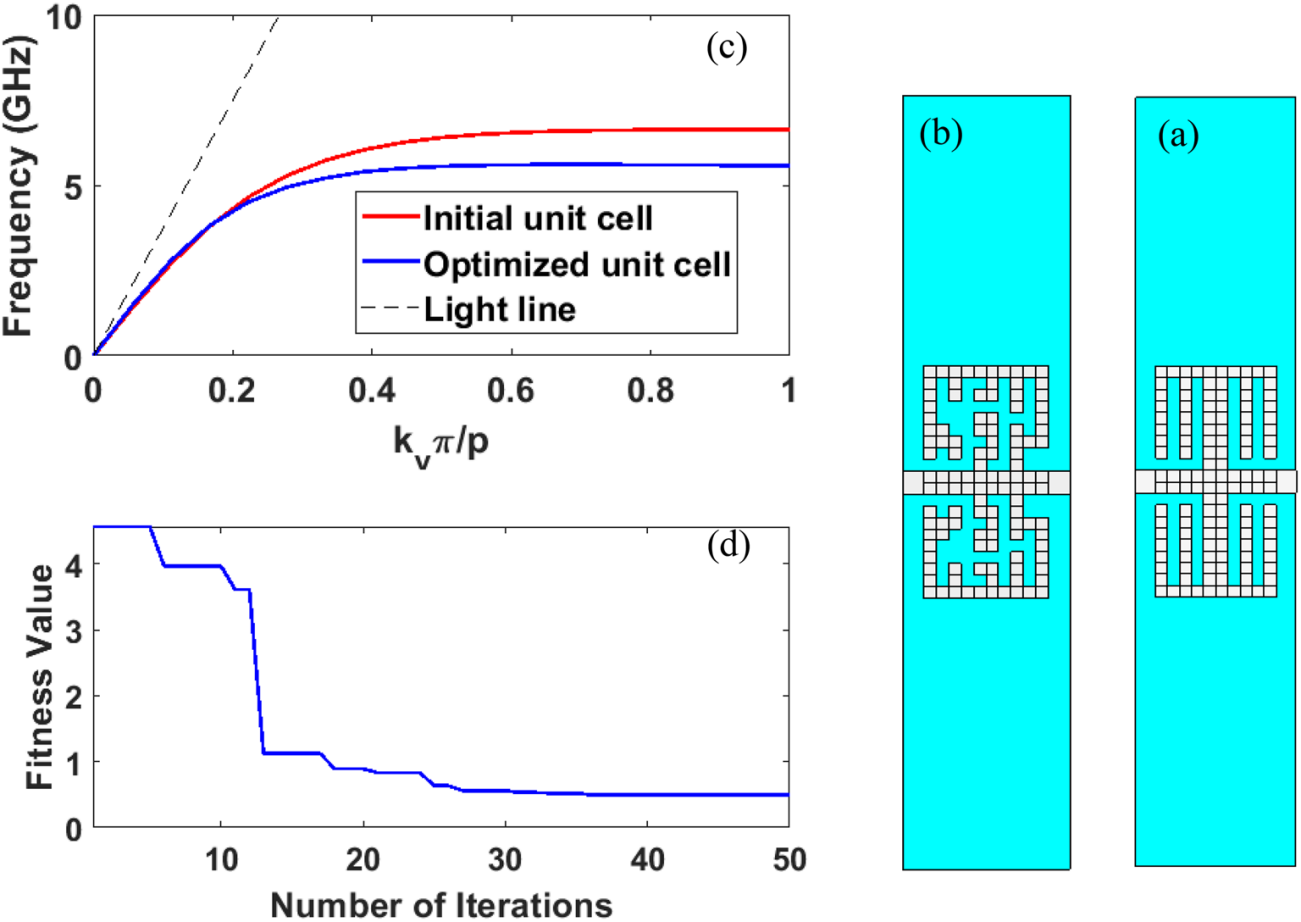
(**a**) Schematic representation of the fence-shaped unit cell as the initial solution according to reference ^14^ in the SSPP structure. (**b**) Schematic representation of the optimized SSPP unit cell, (**c**) comparison of the dispersion curve of the initial unit cell with the optimized unit cell, the parameters in mm are chosen as follows: ${\varvec{p}}_{{\varvec{x}}} = {\varvec{p}}_{{\varvec{y}}} = 0.3$, ${\varvec{p}} = 4$, ${\varvec{w}} = 20$, ${\varvec{l}}_{{\varvec{p}}} = 3$, and ${\varvec{w}}_{{\varvec{p}}} = 6$, (**d**) diagram of the changes of the fitness function values during the optimization iterations.

In the first simulation, the aim of optimization is to obtain a unit cell that has higher desired field confinement than the unit cells that have been presented so far. According to the author’s best knowledge, the fence-shaped unit cell shown in Fig. 4a provides the highest surface wave concentration and, hence, the lowest asymptotic frequency among other unit cells^14^. This unit cell is considered as the initial answer in the optimization process. The asymptotic frequency of the main mode of this unit cell with the dimensions mentioned in Fig. 4 is 6.62 GHz, according to the dispersion curve of Fig. 4c and using simulation in CST MWS. So as to achieve the higher field confinement, the cost function is defined as follows in the k^th^ iteration:

2 $$ fitness^{k} = \mathop \sum \limits_{i = 1}^{N} \mathop {\max }\limits_{{}} \left\{ {f_{i}^{k} - f_{{i_{opt} }} ,0} \right\} $$

where in Eq. (2), N is the number of points on the dispersion curve, $f_{{i_{opt} }}$ is the desired frequency corresponding to the phase of the considered points and $f_{i}^{k}$ is the frequency obtained at the same points in the k^th^ iteration. In this simulation, the number of points on the dispersion curve is considered as N = 5, which is equivalent to $\varphi_{i} = k_{y} \times p = \left\{ {\frac{\pi }{6},\frac{\pi }{3},\frac{\pi }{2},\frac{2\pi }{3},\pi } \right\}$ phases and the optimal frequencies corresponding to these phases are selected as $f_{{i_{opt} }} = \left\{ {4.45, 5.64,5.85,6,6.01 } \right\} {\text{GHz}}$. Given the max term in the cost function defined in Eq. (2), the aim of optimization is bringing the dispersion curve below these points. Since bilateral symmetry is assumed for the unit cell, the number of pixels needed in the optimization is halved and equals 100 with the dimensions mentioned in the caption of Fig. 4. In the optimization, the size of particle’s population is considered as 20 and the number of program’s repetition is considered as 50. The value of the cost function is drawn in terms of optimization repetition steps in Fig. 4d. The shape of the optimized unit cell and its dispersion curve compared to the dispersion curve of the initial unit cell are represented in Fig. 4b and  c, respectively. The asymptotic frequency of the dispersion curve of the optimized unit cell is 5.53 GHz, which is about 1 GHz lower than the asymptotic frequency of the initial dispersion curve.

A comparison between the asymptotic frequencies of the proposed unit cell with some traditional unit cells having the same area is also presented in Table 1. It is found that the asymptotic frequency of the proposed unit cell is much lower than that of the others. The dominant parameter in determining the asymptotic frequency from Eq. (1) is the groove depth $\left( h \right)$. As the groove depth increases, the asymptotic frequency decreases. Since the equivalent groove depth has increased in the proposed structure, the asymptotic frequency has also decreased. Consequently, more concentration of surface waves is obtained.

**Table 1:** The asymptotic frequencies of different SSPP unit cells.

Unit cell Shape	Rectangular	T-shaped	Parallel-arranged	Fence-shaped	Proposed groove
Asymptotic frequency (GHz)	12.75	9.34	8.26	6.62	5.53

**Figure 5:**
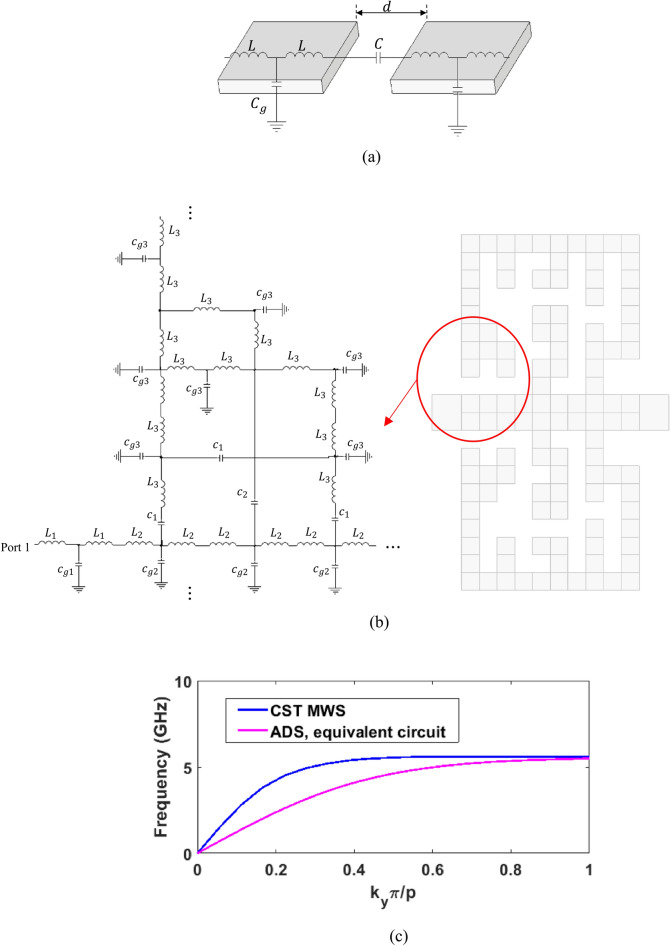
(**a**) The equivalent circuit of pixels, (**b**) The equivalent circuit of a part of the unit cell, (**c**) Dispersion curves of the unit cell obtained with EM simulation and circuit simulation.

For a comprehensive understanding of the propagation characteristic obtained for the proposed unit cell, we have presented an equivalent circuit in Fig. 5. To obtain the equivalent circuit, first, each pixel is modeled with a T-shaped circuit as shown in Fig. 5a, in which $L$ denotes the self-inductance of the metal, $C_{g}$ corresponds to a capacitance with metal at infinity, and $C$ is caused by coupling between two pixels with distance $d$. The value of capacitors and inductors can be calculated by electrostatic simulation of CST MWS and electrostatic formula, respectively^25,26^. The equivalent circuit of the whole unit cell is then obtained by putting together the equivalent circuit of pixels, a part of which is shown in Fig. 5b. The calculated circuit elements are given in Table 2.

**Table 2:** The value of equivalent circuit elements.

Element	$L_{1} \left( {pH} \right)$	$L_{2} \left( {pH} \right)$	$L_{3} \left( {pH} \right)$	$c_{g1} \left( {fF} \right)$	$c_{g2} \left( {fF} \right)$	$c_{g3} \left( {fF} \right)$	$c_{1} \left( {fF} \right)$	$c_{2} \left( {fF} \right)$
Value	40	18	20	46	35	27	8	6

The equivalent circuit was simulated using Keysight Advance Design Systems (ADS) to obtain its impedance matrix. The transmission ABCD matrix is obtained from the impedance matrix and then the propagation constant is calculated from the following equation:

3 $$ \beta = \cos^{ - 1} \left( {\frac{A + D}{2}} \right) $$

The dispersion curves from the equivalent circuit simulated by ADS and the Eigenmode solver of CST MWS are compared in Fig. 5c, which are in acceptable agreement.

**Figure 6:**
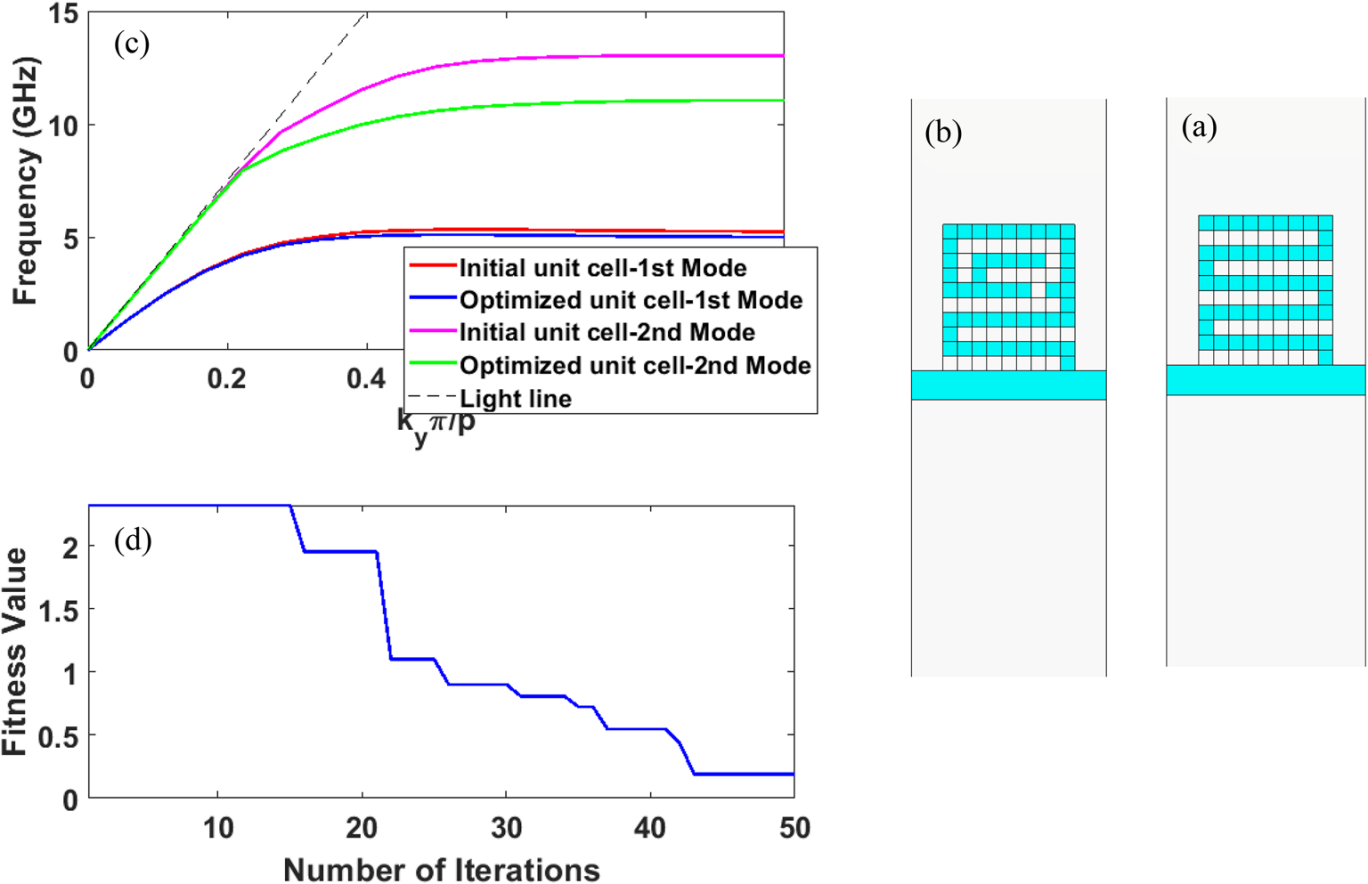
(**a**) Schematic representation of the initial unit cell in the CSSPP structure. According to ^15^, this unit cell is a corrugated slot line in which the interdigital structures are loaded. (**b**) Schematic representation of the optimized CSSPP unit cell, (**c**) comparison of the dispersion curve of the initial unit cell with the optimized unit cell, the parameters in mm are chosen as follows: ${\varvec{p}}_{{\varvec{x}}} = {\varvec{p}}_{{\varvec{y}}} = 0.3$, ${\varvec{p}} = 4$, ${\varvec{w}} = 25$, ${\varvec{l}}_{{\varvec{p}}} = 2.7$, ${\varvec{w}}_{{\varvec{p}}} = 3$, and ${\varvec{w}}_{1} = 0.6$, (**d**) diagram of the changes of the fitness function values during the optimization iterations.

In the second simulation, the unit cell presented in the CSSPP waveguide structure is considered as the initial answer^15^ (Fig. 6a). In this unit cell, the cutoff frequency of the first mode can be controlled by changing the number of IS loaded in the corrugated slot line. Although the mentioned reference does not talk about the effect of the number of IS on the dispersion curve of the next modes, using the similar simulation, one can demonstrate that the cutoff frequency of the second mode also decreases with increasing the IS number, and the dispersion curve of the two modes cannot be controlled independently.

In the optimization of this section, we assume that the dispersion curve of the first mode remains unchanged and only the dispersion curve of the second mode can be changed in a favorable way. This aim is not achieved by changing the number of IS, since that change affects both modes. According to the above-mentioned purpose, the merit function is defined as follows:

4 $$ fitness^{k} = fitness_{1}^{k} + fitness_{2}^{k} $$

5 $$ fitness_{1}^{k} = \mathop \sum \limits_{i = 1}^{N} \left| {f_{i1}^{k} - f_{{i_{opt1} }} } \right| $$

6 $$ fitness_{2}^{k} = \left| {\mathop {\max }\limits_{i} \left\{ {f_{{i_{2} }}^{k} } \right\} - 11} \right| + \mathop {\max }\limits_{{}} \left\{ {8 - f_{2}^{k} \left( {\varphi = \frac{2\pi }{9}} \right),0} \right\} + \mathop {\max }\limits_{{}} \left\{ {f_{2}^{k} \left( {\varphi = \frac{5\pi }{{18}}} \right) - 9.5,0} \right\} $$

The cost function in Eq. (4) consists of two sentences including: $$fitness_{1}^{k}$$ which is related to the first mode and $$fitness_{2}^{k}$$ which is related to the second mode. In Eq. (5), in order to keep the dispersion curve of the first mode unchanged, N points with different phases are selected on the dispersion curve of the first mode of the initial answer. The frequencies related to these points are called $$f_{{i_{opt1} }} $$ and the absolute value of the difference between them with the frequencies obtained in each stage of optimization $$\left( {f_{i1}^{k} } \right) $$ are considered as the cost function. In Eq. (6), which is related to the optimization of the second mode, three terms are take into account: The first term is to ensure that the maximum frequency of the dispersion curve of the second mode does not exceed 11 GHz, while the maximum frequency of the second mode of the initial unit cell is about 13 GHz. The second and third terms are considered so as to make sure that bending of the dispersion curve occurs at the frequency of 8 GHz. With these three terms, it is expected that the surface wave mode between the frequencies of 8 GHz and 11 GHz is created. Also, symmetry is not considered for the unit cell and according to the dimensions mentioned in the caption of Fig. 6, the number of the pixels is 90. Other optimization parameters are considered as before. Figure 6b represents the obtained optimized unit cell. Besides, the dispersion curve related to the first and second modes for the initial and final solutions are shown in Fig. 6c. As can be seen, without a significant change in the dispersion curve of the first mode, the second mode has changed in a favorable way. The convergence curve of the optimization for 50 iterations is also represented in Fig. 6d.

## Experimental results

In order to check the accuracy of the results obtained in the previous section, after designing the unit cells according to the intended dispersion curves, the TLs consisting of these unit cells are designed and simulated; the simulation results are compared with the construction results.

**Figure 7:**
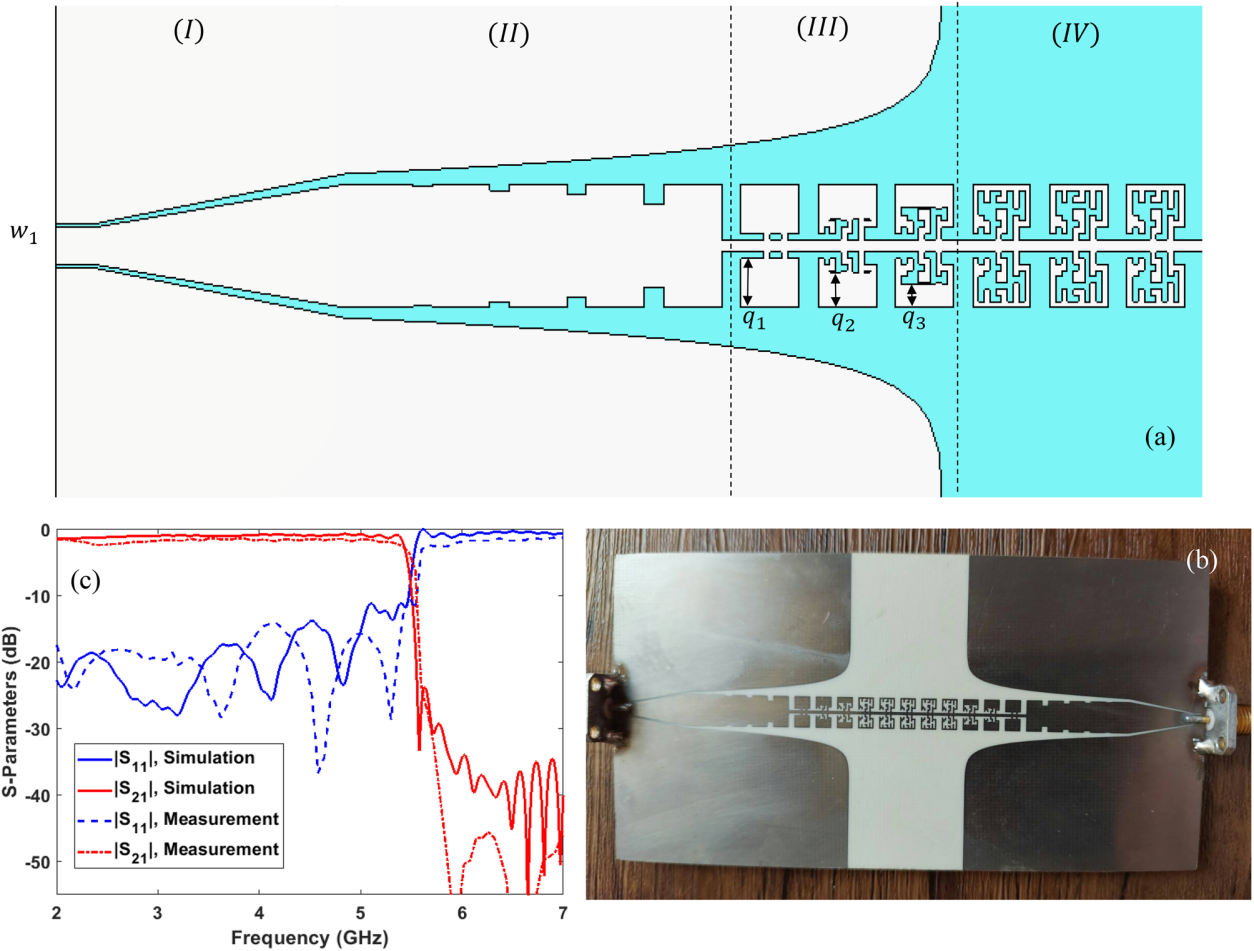
(**a**) Schematic representation of the SSPP TL designed with the unit cell of Fig. 4b, (**b**) A built sample (**c**) Comparison of the simulation and measurement results of the dispersion parameters, the parameters in mm are chosen as follows: ${\varvec{w}}_{1} = 1.8$, ${\varvec{l}}_{1} = 2$, ${\varvec{w}}_{2} = 6$, ${\varvec{l}}_{2} = 13$, ${\varvec{h}}_{1} = 0.1$, ${\varvec{h}}_{2} = 0.3$, ${\varvec{h}}_{3} = 0.5$, ${\varvec{h}}_{4} = 1$, ${\varvec{q}}_{1} = 2.4$, ${\varvec{q}}_{2} = 1.7$, and ${\varvec{q}}_{3} = 1.15$.

Figure 7a shows a part of the SSPP TL, which includes the following four parts: (I) Coplanar waveguide (CPW) for feeding with impedance of 50 ohms at the input; (II) transient part for converting CPW TL into SSPP TL with gradual rectangular unit cell and flaring ground; (III) transient part in converting rectangular SSPP unit cell into the SSPP unit cell of the Fig. 4b; and (IV) SSPP TL with the unit cell of Fig. 4b.

A sample of the designed TL is fabricated (Fig. 7b). The experimental results related to S-parameters are measured using a vector network analyzer (Agilent E8361C model) and compared with the simulation results in Fig. 7c. As can be seen, the results of simulation and measurement are in agreement with each other and the SSPP TL transmits low frequencies to 5.48 GHz based on the dispersion curve.

**Figure 8:**
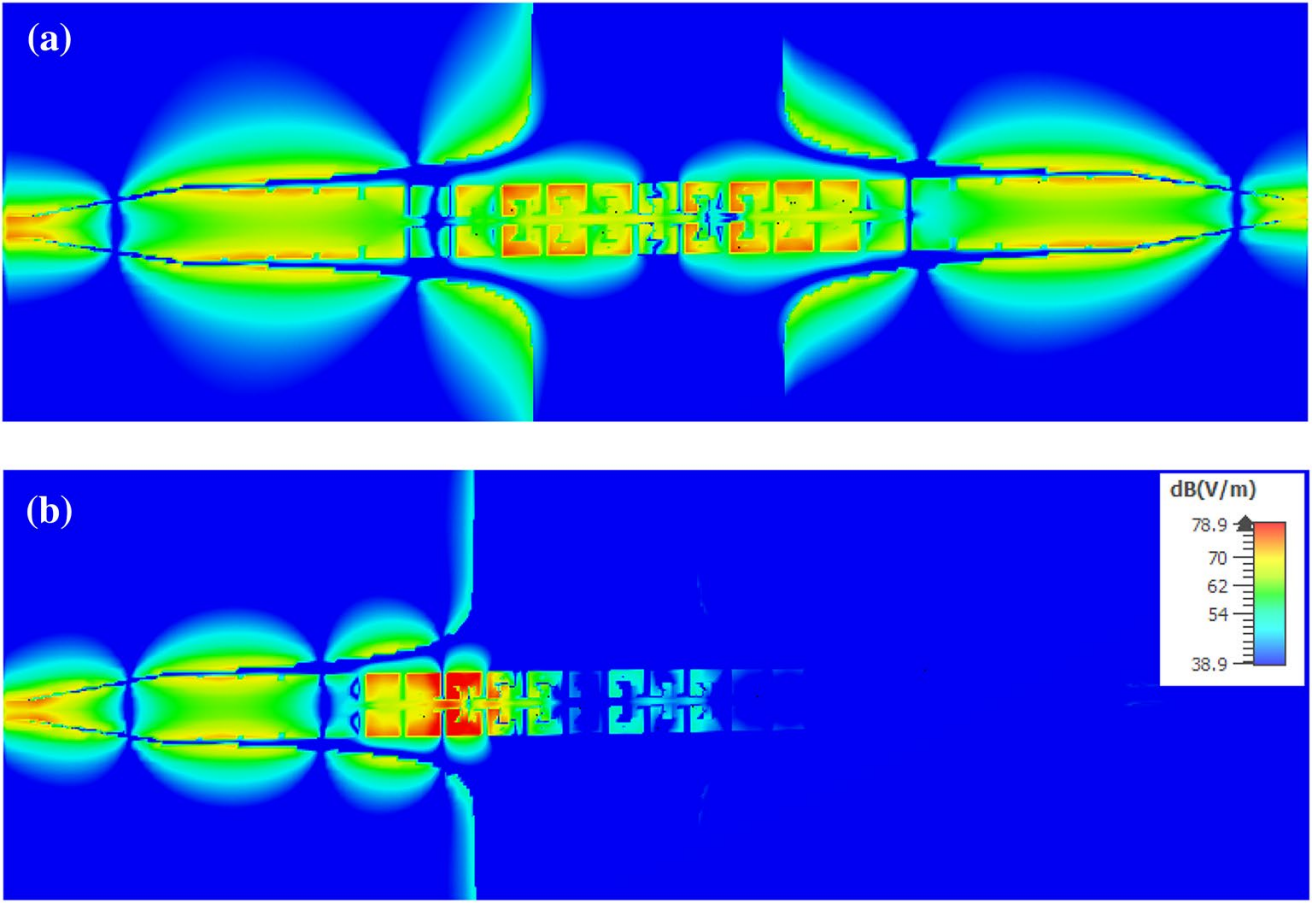
Distribution of the z-component of the simulated electric field in the x–y plane on the upper surface of the SSPP TL at the frequency of (**a**) 4 GHz and (**b**) 6 GHz.

Figure 8 represents the distribution of the z component of the near electric field, in two dimensions of the (x–y) plane and on the upper surface of the structure at two frequencies of 4 GHz and 6 GHz, which is simulated in the CST MWS software. As expected, at the frequency of 4 GHz, the wave propagates effectively in the structure, but at the frequency of 6 GHz, the SSPP unit cells prevent the propagation of the wave.

**Figure 9:**
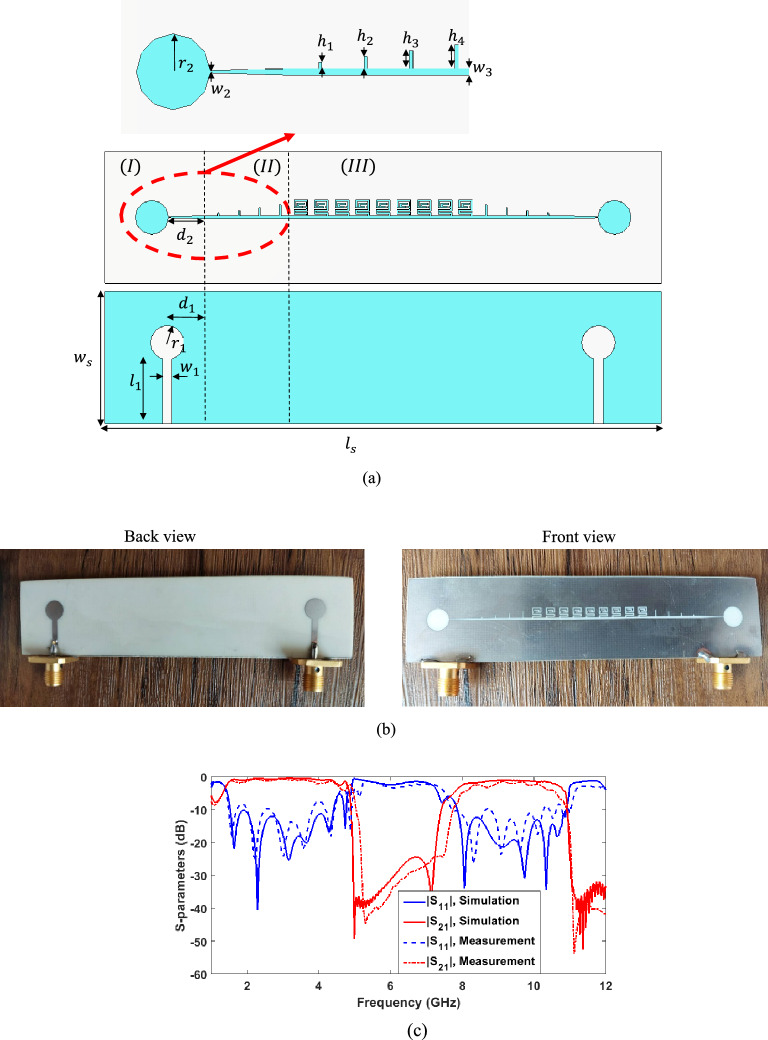
(**a**) Schematic representation of the TL designed with the unit cell of Fig. 6b, (**b**) A built sample (**c**) Comparison of the simulation and measurement results of the scattering parameters, the parameters in mm are chosen as follows: ${\varvec{w}}_{1} = 1.8,$ ${\varvec{l}}_{1} = 12.19,\user2{ d}_{1} = 7.85,\user2{ r}_{1} = 3.2$, ${\varvec{w}}_{2} = 0.16,\user2{ d}_{2} = 7.6,\user2{ r}_{2} = 3.23$, ${\varvec{w}}_{3} = 0.3$, ${\varvec{h}}_{1} = 0.5,$ ${\varvec{h}}_{2} = 1,$ ${\varvec{h}}_{3} = 1.5$, ${\varvec{h}}_{4} = 2,\user2{ l}_{{\varvec{s}}} = 108$, and ${\varvec{w}}_{{\varvec{s}}} = 25$.

Another TL is designed based on the optimized CSSPP unit cell (Fig. 6b), the schematic representation of which is shown in Fig. 9a. This TL is stimulated by a microstrip line placed on the bottom surface of the structure. The microstrip line couples the TEM field to the slot line with gradual changes placed on the upper surface (region I). For proper impedance matching, a circular pad is placed at the end of the microstrip line and a circular slot is placed at the end of the slot line. Four cells with rectangular grooves are placed at the beginning of the CSSPP line, which gradually change the dispersion curve and bring them closer to the dispersion curve of the first two modes of the optimized CSSPP unit cell (region II). In the following, CSSPP unit cells are also placed (unit III). A fabricated sample of this TL is represented in Fig. 9b. Comparison of the simulation and measurement results is represented in Fig. 9c. As predicted by the dispersion curve, this TL provides two pass bands. The first band is related to the first mode, which its lower frequencies are removed owing to the transition of the microstrip line to the slot line and its high pass frequency is up to 4.8 GHz. The second band, according to the dispersion curve of the second mode, starts from the frequency of 7.8 GHz and continues to the frequency of 10.9 GHz $\left( {\left| {S_{11} } \right| < - 10 {\text{dB}}} \right)$. The slight differences between measurement and simulated results can be attributed to the tolerance of the fabrication and imprecise welding of SMA connectors.

**Figure 10:**
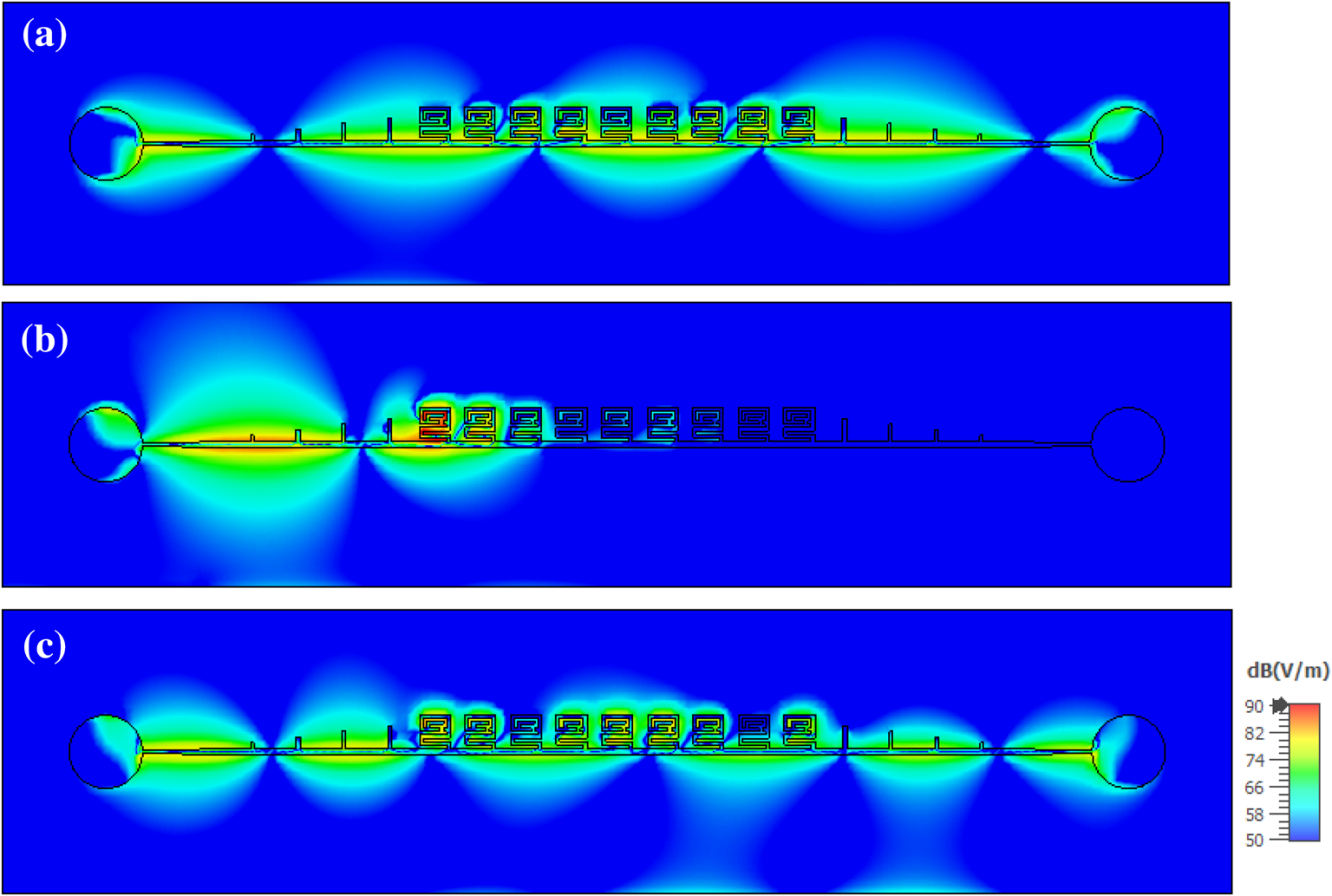
Distribution of the z-component of the simulated electric field in the x–y plane on the upper surface of the CSSPP line at the frequency of (**a**) 4 GHz, (**b**) 6 GHz, and (**c**) 8 GHz.

The distribution of the z component of the electric field above the CSSPP TL at the frequencies of 4, 6, and 8 GHz is represented in Fig. 10. As can be seen from the figure, at the frequencies of 4 GHz and 8 GHz, which correspond to the passband of the first and second modes, respectively, the wave propagates well on the line; however, at the frequency of 6 GHz, which corresponds to the band stop between the two modes, CSSPP unit cells prevent wave propagation.

A comparison between the proposed dual-band SSPP TL with some recent ones is presented in Table 3. The proposed method offers the independent design of the frequency bands of dual-band SSPP TL in thinner layers.

**Table 3:** Comparison of the proposed dual-band SSPP TL with the state-of-the-art works.

References	Frequency bands	Return Loss (dB)	Insertion Loss (dB)	Technology	Thickness (mm)	advantage
^27^	2.65/3.75 GHz Bandwidth: 8.7% and 13.3%	13, 11	1.47, 1.69	Substrate integrated waveguide/SSPP	2.548	Independent control of the passbands using independent choice of geometrical parameters and dielectric materials of multilayer SIW
^28^	1–6.5 GHz/11.5–13 GHz	13, 10	0.6, 2	Microstrip line/SSPP	1	Negative group velocity of the second mode
^29^	Low frequencies to 8.8 GHz/9.7–11.2 GHz	10, 10	Not reported	CPW/SSPP	0.508	Using even higher-order modes
^30^	1–4 GHz/7.5–10.5 GHz	40	0.1	CPW/ SSPP	0.5	High transmission efficiency
This work	1–4.8 GHz/7.8–10.9 GHz	10, 10	1.5, 2.5	Microstrip/slotline/ SPP	0.8	Independent control of frequency bands using topology optimization

## Conclusion

A pixelated topology design method for SSPP unit cell was proposed to achieve the optimal dispersion curve through combining BPSO optimization method and electromagnetic analysis. Unlike the traditional method of controlling the dispersion curve, which is done by changing the geometric parameters, in this method, there is more freedom and even the modes can be controlled independently. Two unit cells were designed using this precise and fully automatic method. For the first design, the optimal dispersion curve of the first mode was considered in such a way that the asymptotic frequency of the unit was about 1 GHz lower than the asymptotic frequency of the most concentrated unit cell that has been introduced so far. In the second design, the dispersion curve of the second mode was optimized independently from the dispersion curve of the first mode. With the designed unit cell, a two-band SSPP TL was obtained, the first band of which was from low frequencies to 4.8 GHz and the second band was from 7.8 to 10.9 GHz. Using this powerful method, it is possible to design unit cells with almost arbitrary dispersion curves for different modes, each of which can be controlled independently, and use them in designing various SSPP TLs.

## Methods

BPSO method was used in order to optimize the topology of the cells. In the BPSO algorithm, which was written in MATLAB, each particle was equivalent to the shape of a unit cell, and the location and velocity of which were updated in the optimization process for each iteration by calling the CST MWS software and calculating the corresponding dispersion curve of the cell. So as to construct SSPP and CSPP lines, RO4003C dielectric film with the dielectric constant of 3.55, loss tangent of 0.0027, thickness of 0.8 mm and copper with conductivity coefficient of ${\upsigma } = 5.8e + 007 {\text{S}}/m$ and thickness of 35 μm were used. To measure S-parameters, vector network analyzer (Agilent E8361C model) was employed.

## Data Availability

The datasets generated and analyzed during the current study are available from the corresponding author on reasonable request.
